# Exome sequencing reveals novel causes as well as new candidate genes for human globozoospermia

**DOI:** 10.1093/humrep/dez246

**Published:** 2020-01-27

**Authors:** M S Oud, Ö Okutman, L A J Hendricks, P F de Vries, B J Houston, L E L M Vissers, M K O’Bryan, L Ramos, H E Chemes, S Viville, J A Veltman

**Affiliations:** 1 Department of Human Genetics, Donders Institute for Brain, Cognition and Behavior, Radboudumc, Nijmegen, The Netherlands; 2 Laboratoire de Diagnostic Génétique, UF3472-génétique de l'infertilité, Hôpitaux Universitaires de Strasbourg, 67000 Strasbourg, France; 3 Institut de Parasitologie et Pathologie Tropicale, EA 7292, Université de Strasbourg, 3 rue Koeberlé, 67000 Strasbourg, France; 4 School of Biological Sciences, Monash University, Clayton, Australia; 5 Department of Gynaecology and Obstetrics, Radboudumc, Nijmegen, The Netherlands; 6 Center for Research in Endocrinology (CEDIE), National Research Council, Department of Endocrinology, Buenos Aires Children’s Hospital, Argentina; 7 Institute of Genetic Medicine, Newcastle University, Newcastle upon Tyne, UK

**Keywords:** globozoospermia, acrosomal hypoplasia, ultrastructure, genetic diagnosis, gene mutation, male infertility, teratozoospermia, exome sequencing, acrosome, consanguinity

## Abstract

**STUDY QUESTION:**

Can exome sequencing identify new genetic causes of globozoospermia?

**SUMMARY ANSWER:**

Exome sequencing in 15 cases of unexplained globozoospermia revealed deleterious mutations in seven new genes, of which two have been validated as causing globozoospermia when knocked out in mouse models.

**WHAT IS KNOWN ALREADY:**

Globozoospermia is a rare form of male infertility characterised by round-headed sperm and malformation of the acrosome. Although pathogenic variants in *DPY19L2* and *SPATA16* are known causes of globozoospermia and explain up to 70% of all cases, genetic causality remains unexplained in the remaining patients.

**STUDY DESIGN, SIZE, DURATION:**

After pre-screening 16 men for mutations in known globozoospermia genes *DPY19L2* and *SPATA16*, exome sequencing was performed in 15 males with globozoospermia or acrosomal hypoplasia of unknown aetiology.

**PARTICIPANTS/MATERIALS, SETTING, METHOD:**

Targeted next-generation sequencing and Sanger sequencing was performed for all 16 patients to screen for single-nucleotide variants and copy number variations in *DPY19L2* and *SPATA16.* After exclusion of one patient with *DPY19L2* mutations, we performed exome sequencing for the 15 remaining subjects. We prioritised recessive and X-linked protein-altering variants with an allele frequency of <0.5% in the population database GnomAD in genes with an enhanced expression in the testis. All identified candidate variants were confirmed in patients and, where possible, in family members using Sanger sequencing. Ultrastructural examination of semen from one of the patients allowed for a precise phenotypic characterisation of abnormal spermatozoa.

**MAIN RESULTS AND ROLE OF CHANCE:**

After prioritisation and validation, we identified possibly causative variants in eight of 15 patients investigated by exome sequencing. The analysis revealed homozygous nonsense mutations in *ZPBP* and *CCDC62* in two unrelated patients, as well as rare missense mutations in *C2CD6* (also known as *ALS2CR11*), *CCIN*, *C7orf61* and *DHNA17* and a frameshift mutation in *GGN* in six other patients. All variants identified through exome sequencing, except for the variants in *DNAH17,* were located in a region of homozygosity. Familial segregation of the nonsense variant in *ZPBP* revealed two fertile brothers and the patient’s mother to be heterozygous carriers. Paternal DNA was unavailable. Immunohistochemistry confirmed that ZPBP localises to the acrosome in human spermatozoa. Ultrastructural analysis of spermatozoa in the patient with the *C7orf61* mutation revealed a mixture of round heads with no acrosomes (globozoospermia) and ovoid or irregular heads with small acrosomes frequently detached from the sperm head (acrosomal hypoplasia).

**LIMITATIONS, REASONS FOR CAUTION:**

Stringent filtering criteria were used in the exome data analysis which could result in possible pathogenic variants remaining undetected. Additionally, functional follow-up is needed for several candidate genes to confirm the impact of these mutations on normal spermatogenesis.

**WIDER IMPLICATIONS OF THE FINDINGS:**

Our study revealed an important role for mutations in *ZPBP* and *CCDC62* in human globozoospermia as well as five new candidate genes. These findings provide a more comprehensive understanding of the genetics of male infertility and bring us closer to a complete molecular diagnosis for globozoospermia patients which would help to predict the success of reproductive treatments.

**STUDY FUNDING/COMPETING INTEREST(S):**

This study was funded by The Netherlands Organisation for Scientific Research (918–15-667); National Health and Medical Research Council of Australia (APP1120356) and the National Council for Scientific Research (CONICET), Argentina, PIP grant 11220120100279CO. The authors have nothing to disclose.

## Introduction

Natural fertilisation occurs upon fusion of a spermatozoon with an oocyte. For the spermatozoon, the acrosome reaction is a crucial step in which the proteolytic contents of the acrosome are released to facilitate penetration through the zona pellucida and to expose key sperm-oocyte binding molecules (Patrat *et al*., 2000). Globozoospermia is a very rare and severe form of infertility and accounts for approximately 0.1% of all cases of male infertility. It is characterised by a round-shaped sperm head and an absence of the acrosome, which explains the inability of these spermatozoa to fertilise an oocyte, and sterility (Dam *et al*. 2007). Globozoospermia can be subdivided into type I (100% acrosomeless round-headed spermatozoa) and type II (>50% acrosomeless spermatozoa). Ultrastructural characterisation has shown that, in addition to pure globozoospermia, some patients have a mixture of acrosomeless spermatozoa and spermatozoa with small or detached acrosomes, which is defined as acrosomal hypoplasia (Zamboni 1987; Baccetti *et al*., 1991; Chemes 2018).

Based on family studies with two or more affected siblings, and the presence of very distinct morphological characteristics of the sperm head, a strong genetic basis was suspected for globozoospermia (Dam *et al*., 2007). Currently, recessive deletions and point mutations in two genes have been firmly identified as responsible for globozoospermia in humans: *DPY19L2* (Harbuz *et al*.*,* 2011; Koscinski *et al*., 2011), accounting for more than 70% of all cases analysed (Ghedir *et al*., 2016; Ray *et al*., 2017), and *SPATA16*, representing less than 2% of the cases (Dam *et al*., 2007; ElInati *et al*., 2016).

Candidate genes for globozoospermia are in pathways involved in the Golgi apparatus function, acrosome formation and the formation as well as the integrity of the acroplaxome between the acrosome and the nuclear membrane during spermiogenesis (Modarres *et al*., 2019). In mice, knockout of at least 48 genes is known to cause globozoospermia or absence of the acrosome (http://www.informatics.jax.org). However, the role of these genes in human globozoospermia remains unknown.

In this study, we aimed to decipher the genetic causes in currently unexplained cases of globozoospermia. For this purpose, we performed whole exome sequencing in 15 globozoospermia patients revealing mutations in seven new genes, of which two have been validated as causing globozoospermia when knocked out in mouse models.

## Materials and Methods

### Patients

This study was approved by the Comité de Protection de la Personne (CPP) at the University Hospital of Strasbourg, France, and the Ethics Review Board of Centro de Investigaciones Endocrinológicas, National Research Council, Buenos Aires, Argentina. All patients gave informed consent. For each of the 16 men, semen analysis to assess sperm concentration and sperm morphology was performed by the IVF clinics treating the patients. Patient GL-3, GL-6, GL-7, GL-10 and GL-11 were diagnosed with globozoospermia type I, and patient GL-1, GL-2, GL-4, GL-8 and GL-9 were diagnosed with type II. The type of globozoospermia was unknown for GL-5, GL-12, GL-13, GL-14 and GL-19. ARG13 was diagnosed with acrosomal hypoplasia. All patients were tested but could not be diagnosed by recurrent homozygous deletions in *DPY19L2* prior to this study. DNA was isolated from a venous blood sample according to routine procedures. DNA from the parents of the sib-pair GL-6 and GL-7 and DNA from two fertile brothers and the mother of GL-11 were also available for this study.

### Pre-screening for mutations in genes known for globozoospermia using targeted sequencing

Prior to exome sequencing, absence of AZF deletions, aneuploidies, *DPY19L2* deletions and mutations in *SPATA16* were excluded using targeted sequencing of a panel of male infertility genes as previously described (Oud *et al*., 2017). As analysis of *DPY19L2* is complicated by the presence of pseudogenes, additional Sanger sequencing of the gene was used to exclude *DPY19L2* point mutations, using a protocol described earlier (Ghedir *et al*. 2016) with several changes related to laboratory-specific set up (Supplementary Tables SI and SII).

**Table I:** Results targeted NGS and Sanger sequencing of *DPY19L2*.

Sample	Targeted NGS	Sanger gene sequencing
GL-6	Heterozygous deletion	No pathogenic variants
GL-7	Heterozygous deletion	No pathogenic variants
GL-14	Heterozygous deletion	Chr12(GRCh37):g.64038271C>G NM_173812.4:c.715G>C p.(Gly239Arg)

### Exome sequencing and bioinformatic analysis

For 15 of 16 patients, exome sequencing was performed. GL-1 to GL-13 and GL-19 exome library preparation was done with Agilent SureSelect Human All Exon V5 (Agilent Technologies, Santa Clara, CA), and sequencing was performed by Genome Diagnostics Nijmegen (https://www.genomediagnosticsnijmegen.nl) using the NextSeq 500 platform (Illumina, San Diego, CA). The ARG13 sample was enriched with the Illumina TruSeq Rapid Exome Capture Kit (Illumina) and sequenced on the NextSeq 500 platform by the Institute of Genetic Medicine at Newcastle University in Newcastle, UK. Read mapping and variant and copy number variation (CNV) calling for all samples were performed using the in-house pipeline of the Radboudumc Genome Technology Centre. Homozygosity calling was performed using RareVariantVis (Stokowy *et al*., 2016).

### Filtering

We selected only single-nucleotide variants (SNVs) that (i) were present in at least five variant sequencing reads and (ii) were present in more than 15% of reads covering that locus. Next, we excluded all variants with an allele frequency ≥ 0.5% in GnomAD, dbSNP and our local database containing >15 000 alleles and selected only non-synonymous and splice variants. For the three affected sib-pairs, we selected only those variants shared by both brothers. We focussed on an autosomal recessive or X-linked mode of transmission and looked for autosomal homozygous, compound heterozygous and X-linked variants. We then only selected variants in genes known to be expressed at elevated levels in the testis (*n* = 2237); indicative protein localisation was obtained from the Human Protein Atlas version 18.1 (Uhlen *et al*., 2015).

All remaining variants were curated for variant quality using manual inspection of the BAM file in the Integrative Genomics Viewer 2.4 (http://software.broadinstitute.org/software/igv/) resulting in the elimination of likely false-positive calls. All variants were classified according to the American College of Medical Genetics and Genomics (ACMG) and the Association for Molecular Pathology (AMP) 2015 guidelines (Richards *et al*., 2015). All prioritised variants were confirmed with Sanger Sequencing.

### Immunohistochemical localisation of *ZPBP* during spermatogenesis

Testis material was obtained with consent from an otherwise healthy male presenting with unexplained testicular pain requiring orchidectomy as described previously (Kennedy *et al*., 2004). The testis biopsies were fixed in Bouin’s solution for 5 h at room temperature (RT), processed and cut into 5-μm sections. Sections were stained overnight at 4°C using a 4-μg/ml mouse α-ZPBP1 antibody (F-12: sc-393 152; Santa Cruz Biotechnology, USA), after which donkey anti mouse-488 secondary antibody was added for 1 h at room temperature. Next, nuclei were counterstained with TO-PRO 3 and imaged with an SP8 confocal microscope as previously described (Dunleavy *et al*., 2017).

### Electron microscopy

A semen sample from patient ARG13 was diluted 1:5 with 0.1 M phosphate buffer (0.1 M, pH 7.4), pelleted by centrifugation and fixed in 3% buffered-glutaraldehyde, post-fixed in 2% osmium tetroxide and embedded in Epon-Araldite resin. Thin sections were obtained with a Pelco diamond knife in an RMC MT-7000 ultra-microtome, mounted on 300-mesh copper grids, double-stained with uranyl acetate and lead citrate and examined and photographed in a Zeiss EM109T electron microscope.

## Results

### Pre-screening for known causes of globozoospermia using targeted sequencing

First, all 16 patients (described in Table III) were pre-screened for deletions and/or mutations in *DPY19L2* and *SPATA16,* using a combination of targeted next-generation sequencing (NGS) and Sanger sequencing to detect point mutations and CNVs in these two genes and other known infertility genes (Oud *et al*., 2017). No pathogenic variants were identified in *SPATA16*. However, in patients GL-6, GL-7 and GL-14, we identified heterozygous *DPY19L2* deletions and only a likely pathogenic missense variant in GL-14, but not in GL-6 and GL-7 (Table I). Given that no conclusive diagnoses were obtained for 15 out of 16 patients by looking at *SPATA16* and *DPY19L2*, exome sequencing was performed for these patients.

### Exome sequencing to find new causes of globozoospermia

Globozoospermia is an extremely rare form of isolated primary infertility, and therefore, we prioritised ultra-rare (<0.5% allele frequency in population databases) SNVs segregating in a recessive or X-linked manner in genes with elevated expression in the testis (Supplementary Table SIII). After filtering and validation by Sanger sequencing, a total of 14 variants in 12 genes remained in 8 patients (Table II; Supplementary Table SIV), in addition to the *DPY19L2* variant in GL-14. No high-confidence variants were found after filtering in patients GL-5, GL-10 or GL-19 or in both sib-pairs GL-6/GL-7 and GL-8/GL-9. For the 12 genes in which we found plausible variants, we then checked the existence of knockout mouse models for these genes, as well as a known role in acrosome formation or function.

**Table II:** **Overview of all prioritized and validated variants identified in this study.** A detailed description of all variants and pathogenicity prediction scores are available in Supplementary Table SIV.

Patient	Gene	Variant	Zygosity	GnomAD variant frequency (population with highest frequency)*	Variant classification according to the ACMG/AMP 2015 guideline	Mouse model	Link to acrosome biology	Conclusion
GL-1 GL-2	*GGN*	p.(Gly424Alafs*65)	Homozygous	0.00% (SAS: 0.01%)	Likely pathogenic	Yes, meiotic arrest (Jamsai et al. 2013)	No	Possibly causative
GL-3	*DNAH17*	p.(Arg944Trp) p.(Phe2594Ile)	Heterozygous Heterozygous	0.15% (NFE: 0.24%) Absent	Uncertain significance Uncertain significance	Yes, male infertility (Dickinson et al. 2016)	No	Possibly causative
GL-3	*MAGEA3*	p.(Leu201Phe)	Hemizygous	0.00% (SAS: 0.01%)	Uncertain significance	No	No	Unlikely causative
GL-4	*C2CD6* (*ALS2CR11*)	p.(His113Arg)	Homozygous	Absent	Uncertain significance	No	Yes (Wang et al. 2015)	Possibly causative
GL-11	*ZPBP*	p.(Gln311*)	Homozygous	Absent	Likely pathogenic	Yes, globozoospermia (Lin et al. 2007)	Yes (Lin et al. 2007)	Likely causative
GL-11	*TM4SF19*	p.(Val68Leu)	Homozygous	0.02% (OTH: 0.15%)	Uncertain significance	No	No	Unlikely causative
GL-12	*CCIN*	p.(Gly285Ser)	Homozygous	0.00% (NFE: 0.01%)	Uncertain significance	No	Yes (Lecuyer et al. 2000)	Possibly causative
GL-13	*CCDC62*	p.(Gln148*) p.(His283Tyr)	Homozygous Homozygous	Absent 0.01% (NFE: 0.01%)	Likely pathogenic Uncertain significance	Yes, globozoospermia (Li et al. 2017)	Yes (Li et al. 2017)	Likely causative
GL-13	*CCDC73*	p.(Leu224Phefs*11)	Homozygous	0.00% (NFE: 0.00%)	Uncertain significance	Yes, no infertility (Khan et al. 2018)	No	Unlikely causative
GL-13	*NRIP3*	p.(Ile132Asn)	Homozygous	Absent	Uncertain significance	No	No	Unlikely causative
GL-13	*ATP8A2*	p.(Arg778Gln)	Homozygous	0.04% (ASJ: 0.10%)	Uncertain significance	No	No	Unlikely causative
GL-14	*DPY19L2*	N/A p.(Gly239Arg)	Heterozygous Hemizygous	Unknown Absent	Pathogenic Likely pathogenic	Yes, globozoospermia (Koscinski et al. 2011; Harbuz et al. 2011)	Yes (Koscinski et al. 2011; Harbuz et al. 2011)	Likely causative
ARG13	*C7orf61*	p.(Glu87Argfs*46)	Homozygous	Absent	Likely pathogenic	No	Yes (Behrouzi et al. 2013)	Possibly causative

^*^GnomAD variant frequency was downloaded from: http://gnomad.broadinstitute.org/ (version 2.1). SAS: South Asian, NFE: Non-Finnish European, OTH: Other, ASJ: Ashkenazi Jewish.

### Homozygous loss-of-function variants in genes implicated in globozoospermia in mice

**Figure 1:**
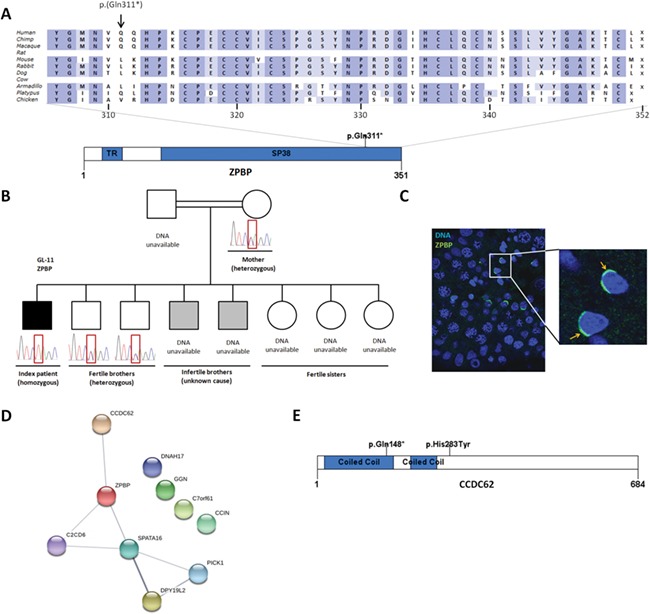
**Mutations in zona pellucida binding protein (*ZPBP*) and in coiled-coil domain containing 62 (*CCDC6*2).** **A** Homozygous nonsense mutation in *ZPBP* observed in the exome data of patient GL-11. The nonsense mutation likely leads to a truncated protein and disrupts the conserved Sp38 domain. **B** Segregation analysis in the family of GL-11. Sanger sequencing was performed on all available DNA samples. **C** Immunocytochemistry of ZPBP (green) in healthy human testis material. ZPBP localises to the acrosome of spermatids (arrow). **D** STRING Network analysis of known and candidate genes for globozoospermia (STRING version 10.5). **E** Homozygous nonsense mutation (c.442C > T; p.Gln148*) and missense mutation (c.847C > T; p.His283Tyr) in *CCDC62* observed in the exome data of patient GL-13. Protein domains were predicted by SMART (http://smart.embl-heidelberg.de/). SP38 = zona-pellucida-binding protein (InterPro: IPR010857). The ortholog alignment was made by Alamut Visual version 2.10 (http://www.interactive-biosoftware.com).

Firstly, we found likely pathogenic variants in two genes already known for globozoospermia when mutated in mice. Our filtering strategy revealed that patient GL-11, who was conceived from first-degree-cousin parents, carries a homozygous nonsense mutation (c.931C > T; p.(Gln311*)) in zona pellucida binding protein (*ZPBP*) (Fig. 1A; Table II; Supplementary Table SIV). Knockout of this gene in mice causes globozoospermia (Lin *et al*., 2007). The patient mutation is predicted to result in a truncated protein lacking the 40 last amino acids of the conserved SP38 domain (Fig. 1A). The variant is located in a homozygosity region of approximately 24 Mb on chromosome 7 (Supplementary Fig. S1) and is completely absent in all population databases. Two brothers of GL-11 are also infertile, but of an unknown aetiology, and DNA is unavailable. DNA was, however, available from two fertile brothers and the mother of GL-11. All three individuals were heterozygous carriers of the nonsense variant (Fig. 1B). Using immunostaining, we confirmed acrosome localisation of the protein in human spermatids (Fig. 1C). Of interest, *ZPBP* forms part of the same gene interaction network with known globozoospermia genes *SPATA16* and *DPY19L2* (Fig. 1D), thus suggesting they are mechanistically linked. Collectively therefore, these data suggest that the aetiology of globozoospermia in GL-11 was a homozygous nonsense mutation in *ZPBP.*

Patient GL-13 showed five homozygous variants in four genes (Table II; Supplementary Table SIV). Four of these are variants of uncertain significance: p.(His283Tyr) in coiled-coil domain containing 62 (*CCDC62*), p.(Leu224Phefs*11) in coiled-coil domain containing 73 (*CCDC73*), p.(Ile132Asn) in nuclear receptor-interacting protein 3 (*NRIP3*) and p.(Arg778Gln) in ATPase phospholipid transporting 8A2 (*ATP8A2*). Of note, the knockout mouse model for *Ccdc73* does not show any phenotype and males are fertile (Kahn *et al*., 2018). The fifth variant identified is a nonsense variant (c.442C > T; p.(Gln148*)) in *CCDC62* (Fig. 1E). The variant is located in a stretch of homozygosity of approximately 9 Mb on chromosome 12 (Supplementary Fig. S2). A homozygous nonsense mutation in *Ccdc62,* called repro29, was shown to cause acrosome defects similar to globozoospermia (Li *et al*., 2017). Similar to the finding in mice, CCDC62 localises to the acrosome in human spermatozoa (Li *et al*., 2017). The premature stop codon is located before the missense variant and is predicted to cause nonsense-mediated decay resulting in an absence of protein. The pathogenicity classification and function of the gene make the homozygous nonsense variant in *CCDC62* the most likely cause of globozoospermia in this patient.

**Figure 2:**
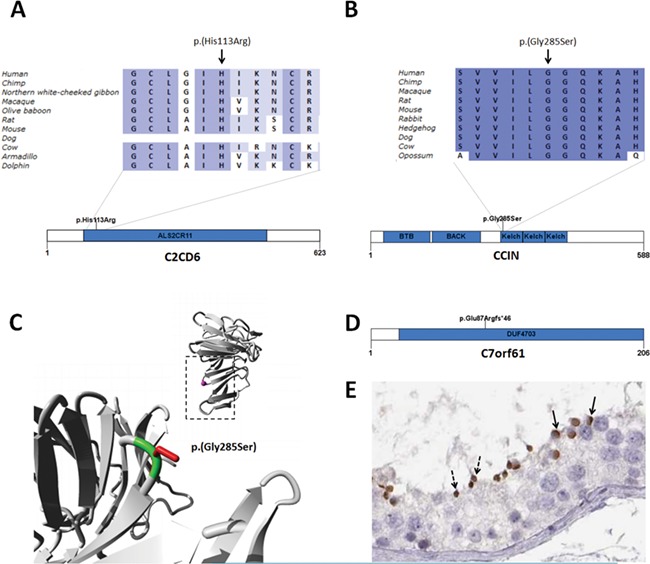
**Mutations in candidate genes for globozoospermia.** **A** Homozygous missense mutation in *C2CD6* in patient GL-4 affecting a conserved amino acid in the ALS2CR11 domain. **B** Homozygous missense mutations in *CCIN* detected in GL-12 affecting a conserved amino acid in a Kelch domain. **C** Structural model of a homologous Kelch domain was used to model the effect of the mutation on protein structure. In the top right corner, an overview of the domain is represented as a ribbon. The side chain of the mutated residue is coloured in magenta and shown as a small ball. In the lower left corner, a close-up of the mutation is shown (region indicated by rectangle). The protein domain is coloured in grey; the side chains of both the wild-type and mutant residues are shown and coloured green and red, respectively. The structural model and images were made by HOPE (http://www.cmbi.umcn.nl/hope/) (Venselaar *et al*., 2010). **D** Homozygous frameshift mutation in *C7orf61* detected in patient ARG13. **E** C7orf61 is localised in the acrosomes of round spermatids (solid arrow) and spermatozoa (dashed arrow). Image credit: Human Protein Atlas. This image is adapted from imid:20265234 available from v18.1.proteinatlas.org (Uhlen *et al*., 2015). Protein domains were predicted by SMART (http://smart.embl-heidelberg.de/). ALS2CR11: amyotrophic lateral sclerosis 2 candidate 11 (InterPro: IPR031462). BTB: broad-complex, tramtrack and bric a brac (InterPro: IPR000210). BACK: BTB and C-terminal kelch (InterPro: IPR011705). Kelch: Kelch repeat type 1 (InterPro: IPR011705). DUF4703: domain of unknown function (InterPro: IPR031534). Ortholog alignments were made by Alamut Visual version 2.10 (http://www.interactive-biosoftware.com).

### Identification of variants in genes with a link to acrosome biology

We also identified mutations in three genes with a known link to acrosome biology, but for which no knockout mouse model is currently available.

In patient GL-4, a homozygous missense variant (c.338A > G; p.(His113Arg)) was found in C2 calcium-dependent domain-containing 6 (*C2CD6*, also known as *ALS2CR11*) (Fig. 2A; Table II; Supplementary Fig. S3Supplementary Table SIV). The variant has not been recorded before in population databases. The missense variant is located in the conserved C2 domain (InterPro: IPR000008) which has an important function in calcium-dependent phospholipid binding and targeting proteins to cell membranes (Nalefski and Falke, 1996). *C2CD6* is detected throughout spermatogenesis (Guo *et al*., 2018) and interacts with *SPATA16* and *ZPBP* (Fig. 1D).

**Figure 3:**
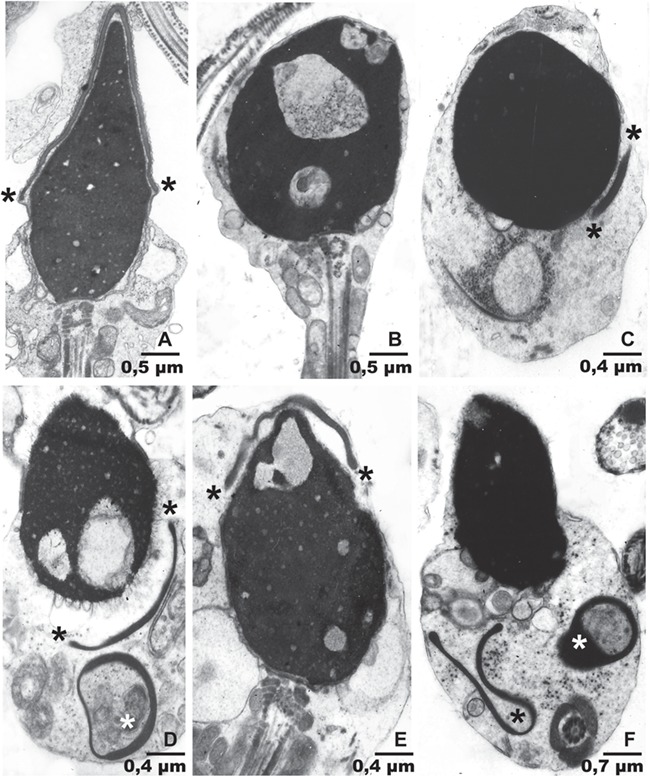
**Electron microscopy in patient ARG13.** **A** Normal sperm head from a fertile individual. Note its elongated shape with a 2.4/1 length to transversal diameter ratio. The thin, dense acrosome covers 2/3 of the head surface. The extension and/or location of the acrosome is indicated by asterisks. **B**–**F** Sperm from ARG13. B: A rounded acrosomeless head depicts three large chromatin rarefactions. C: This round head with normal chromatin has an extremely small acrosome in its lower right corner. D: Ovoid sperm head with two large chromatin rarefactions and a small detached acrosome in its lower right corner. Another small, ring-like acrosome is present underneath. E: The elongated head has a big chromatin rarefaction in its cranial aspect and is covered by a small, hypoplastic acrosome. F: An ovoid sperm head with normal chromatin is devoid of a normally positioned acrosome. Two acrosomal structures lay free in the cytoplasm underneath the head. Panel magnification is indicated by length of the bars.

In patient GL-12, a homozygous missense variant (c.853G > A; p.(Gly285Ser)) was found in Calicin (*CCIN*) (Fig. 2B; Table II; Supplementary Fig. S4; Supplementary Table SIV). The variant is located in a conserved region of the Kelch repeat type 1 domain (InterPro: IPR006652). A 3D structure of a homologous Kelch domain is available (PDB: 2XN4), and structural modelling revealed that the variant is located on the surface of the Kelch domain, which is important for binding of other molecules such as actin filaments (Fig. 2C). The differences between the wild-type and mutant residues may influence the interaction with other molecules or other parts of the molecule. The torsion angles for the mutated residue may not be flexible enough, which can force the local backbone into an incorrect conformation disturbing the local structure (Fig. 2C). In humans, CCIN binds actin in the acrosomal region of round spermatids and localises to the post-acrosomal region of elongated spermatids (Lecuyer *et al*., 2000).

**Figure 4:**
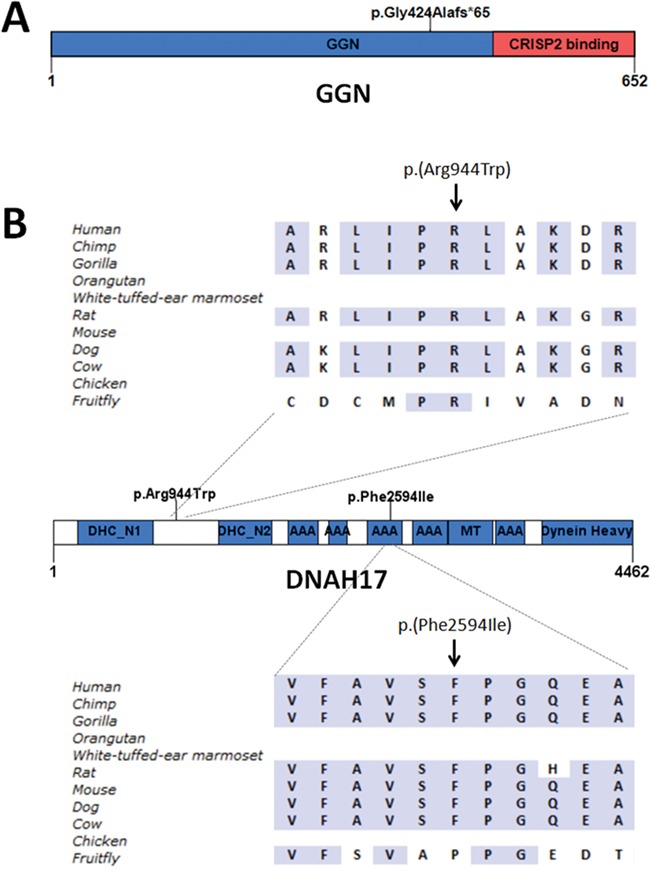
**Mutations in genes not directly linked to globozoospermia.** **A** Homozygous frameshift mutation in *GGN* in brothers GL-1 and GL-2. **B** Heterozygous missense mutations in *DNAH17* in GL-3. GGN: gametogenin. DHC_N1: dynein heavy chain, N-terminal region 1. DHC_N2: dynein heavy chain, N-terminal region 2. AAA: ATPase associated with diverse cellular activities domain (hydrolytic ATP binding site of the dynein motor region). MT: microtubule-binding stalk of dynein motor. Dynein Heavy: dynein heavy chain region D6 P-loop domain.

Patient ARG13 carries a homozygous frameshift variant (c.259del; p.(Glu87Argfs*46)) in chromosome 7 open reading frame 61 (*C7orf61*) (Fig. 2D; Table II; Supplementary Fig. S5; Supplementary Table SIV). The variant is absent from population databases. Normal spermatozoa from fertile men have 3–5-μm-long heads with dense, compact chromatin. The acrosome closely attaches to the head and covers approximately 2/3 of its cranial surface (Fig. 3A). In contrast to this, more than 95% of spermatozoa from patient ARG13 possessed conspicuous head-shaped and acrosomal anomalies. Many heads were close to spherical with absent or minute acrosomes (globozoospermic). Others were roundish or irregularly ovoid and had small acrosomes, not well attached to the nuclear surface or completely disengaged from it (acrosome hypoplasia, Fig. 3B–F). Additionally, there were frequent defects in chromatin compaction, as indicated by nuclear pseudo-vacuoles containing lightly granular or amorphous material (Fig. 3B–F). The function of *C7orf61* remains largely elusive. The protein was detected in the insoluble fraction of human sperm cells including the nucleus, sperm tail and perinuclear theca (Behrouzi *et al*., 2013). Consistent with this finding, the protein is localised to the peri-acrosomal region of spermatids (Fig. 2E). The data suggest that C7orf61 protein may therefore play an important role in acrosome formation and nucleus shaping during spermiogenesis and can be considered a novel candidate gene for acrosomal hypoplasia.

### Variants in genes without an obvious link to globozoospermia or acrosome biology

Finally, we also found ultra-rare variants in genes that have a link with male infertility but not globozoospermia. Specifically, brothers GL-1 and GL-2 are both homozygous for a frameshift mutation (c.1271del; p.(Gly424Alafs*65)) in the first coding exon of Gametogenetin (*GGN*) (Fig. 4A; Table II; Supplementary Table SIV). Both brothers carry large overlapping stretches of homozygosity (both approximately 7 Mb) on chromosome 19 containing *GGN* (Supplementary Fig. S6). Homozygous knockout of *Ggn* in mice causes pre-implantation embryonic lethality, and heterozygous mice showed deficient double-strand break repair during male meiosis (Jamsai *et al*., 2013). As this early lethality precluded an analysis of the consequences of *GGN* ablation in spermatids, the possibility remains that it has a role in acrosome biology.

Finally, patient GL-3 carries two heterozygous missense variants in dynein axonemal heavy chain 17 (*DNAH17*) (Fig. 4B; Table II; Supplementary Table SIV). Both variants were classified as Variant of Unknown Significance. The same patient also carries a hemizygous variant of uncertain significance (c.601C > T; p.(Leu201Phe)) in *MAGEA*3. The function of this protein is not known to be related to globozoospermia. It remains uncertain whether variants in *DNAH17* or *MAGEA*3 cause globozoospermia.

## Discussion

Globozoospermia is commonly caused by recessive deletions of *DPY19L2*, but a significant fraction of all patients remain undiagnosed (Dam *et al*., 2007; Harbuz *et al*., 2011; Koscinski *et al*., 2011). In this study, we aimed to identify new genes involved in globozoospermia by exome sequencing in unexplained globozoospermia cases. Our analysis revealed likely pathogenic variants in two known mouse globozoospermia genes and variants in five novel candidate genes for human globozoospermia (Tables II and III).

**Table III:** Overview of familial and clinical data.

Patient	Globozoospermia type	Candidate gene	Ethnicity	Consanguinity	Fertility in family members	Sperm conc. (sperm/ml) reported by IVF clinic	ICSI result
GL-1	2	*GGN*	Turkish	Yes (unknown degree)	1 brother with globozoospermia (GL-2), 1 fertile brother with 4 children)	< 2 million	3xICSI: 25% fertilization, no success
GL-2	2	*GGN*	Turkish	Yes (unknown degree)	1 brother with globozoospermia (GL-1, 1 fertile brother with 4 children)	2 million	2xICSI: no fertilization
GL-3	1	*DNAH17*	French (Mulhouse)	No	2 fertile sisters	10 million	6xIUI: no success 1xIVF with donor sperm: child is born
GL-4	2	*C2CD6*	Moroccan	Yes (mother is the niece of the father)	3 brothers and 2 sisters, all fertile	Not available	No ICSI
GL-5	Unknown	None	Unknown	No	Unknown	Unknown	Unknown
GL-6	1	None	French (Dijon)	No	1 brother with globozoospermia (GL-7), 1 infertile half-sister	38 million	1xICSI: healthy girl was born
GL-7	1	None	French (Dijon)	No	1 brother with globozoospermia (GL-6), 1 infertile half-sister	109 million	2xICSI: no success
GL-8	2	None	French (Lille)	No	2 brothers with globozoospermia including GL-9	Unknown, 95% atypical sperm	1xICSI: 25% fertilization, 1 child was born
GL-9	2	None	French (Lille)	No	2 brothers with globozoospermia including GL-8	Unknown, 98% atypical sperm	1xICSI: 25% fertilization, 1 child was born, 1 additional pregnancy
GL-10	1	None	Not known	No	Unknown	Unknown	Sperm donation: twins were born
GL-11	1	*ZPBP*	Moroccan	Yes (unknown degree)	2 infertile brothers	52 million	2 children
GL-12	Unknown	*CCIN*	Lebanese	Yes (unknown degree)	3 infertile brothers, 1 fertile sister	< 2 million	1xICSI: pregnancy (2011)
GL-13	Unknown	*CCDC62*	Lebanese	Yes (unknown degree)	1 fertile sister, 1 infertile male cousin	32 million	Unknown
GL-14	Unknown	*DPY19L2*	USA	No	Unknown	4 million	1xICSI: 1 pregnancy from frozen embryo transfer, healthy boy was born
GL-19	Unknown	None	The Netherlands (indicated as Caucasian)	No	No affected brothers	180 million	6xIUI: no pregnancy 1xIVF: no fertilization 3xICSI: 1 healthy girl was born
ARG13	Acrosomal hypoplasia	*C7orf61*	Argentina	Yes (unknown degree)	Unknown	Unknown, 95% atypical sperm	Unknown

### Novel genetic causes of globozoospermia

We identified two likely pathogenic homozygous nonsense mutations, which most probably lead to a truncated protein (*ZPBP;* c.931C > T; p.(Gln311^*^)) or nonsense-mediated mRNA decay (*CCDC62;* c.442C > T; p.(Gln148^*^)), in genes previously known to cause globozoospermia when mutated in mice (Lin *et al*., 2007; Li *et al*., 2017).

Similar to the findings in mice, ZPBP (Fig. 1C) and CCDC62 (Li *et al*., 2017) are both localised to the acrosome in human sperm. Indeed, sperm cells from the *Zpbp1* knockout mouse have severely disorganised acrosomes, abnormal nuclear shape, excessive cytoplasm and coiled sperm tails (Lin *et al*., 2007). Mutations in *ZPBP* have been described before in patients with sperm head defects (Yatsenko *et al*., 2012), but it remains unclear if these patients suffered from globozoospermia. Also, the involvement of the described missense and splice mutations in disease has not clearly been demonstrated.

Loss of *Ccdc62* in mice leads to fragmentation of the acrosome in the maturation phase of acrosome development and abnormal bending and cytoplasmic retention around the sperm head (Li *et al*., 2017). *CCDC62* contains coiled-coil domains similar to *PICK1* and *GOPC,* whose knockout mouse models display also globozoospermia (Yao *et al*., 2002; Xiao *et al*., 2009). CCDC62 was shown to interact with GOPC, whereas no interaction between CCDC62 and PICK1 was observed (Wang *et al*., 2015). Interestingly, in humans, *CCDC62* was shown to be co-expressed with *ZPBP*. However, the exact role of *CCDC62* in human globozoospermia remains unclear. Unfortunately, no sperm or testis material of the patient with the *CCDC62* mutation was available to perform further functional studies.

Our patient with a homozygous *ZPBP* mutation had two children after intra-cytoplasmic sperm injection (ICSI) treatment: a healthy baby girl and a boy with cardiofaciocutaneous (CFC) syndrome. CFC syndrome is caused by *de novo* mutations in genes involved in the RAS/MAPK signalling pathway in most cases (Pierpont *et al*., 2014). Whereas a contribution of the variant in *ZPBP* to the son’s phenotype cannot be excluded, a link between *ZPBP* and the RAS/MAPK pathway has not been reported before.

### Novel candidate genes for globozoospermia

In addition to mutations in genes already linked to globozoospermia in mice, we identified variants in novel genes *C2CD6* (c.338A > G; p.(His113Arg))*, CCIN* (c.853G > A; p.(Gly285Ser)) and *C7orf61* ((c.259del; p.(Glu87Argfs*46)), with a clear link to acrosome biology. All three genes are expressed at elevated levels in the testis. The involvement of *C2CD6* in the acrosome formation and function is based on its interaction with SPATA16 and ZPBP. CCIN and C7orf61 have an acrosomal localisation (https://www.proteinatlas.org/). In addition, CCIN is known to bind to actin. Therefore, it could be involved in the transport of acrosomal vesicles from the Golgi apparatus to the apical region of the sperm head during acrosome biogenesis. Mouse models to morphologically assess sperm function and male fertility, however, are not yet available. Hence, in order to learn more about the role of these genes in globozoospermia, the generation of such animal models would be of great value.

We also identified variants in genes for which no data are yet available concerning their function in the acrosome formation. GL-1 and GL-2 are brothers and share homozygous frameshift mutations in *GGN.* While data support a role for *Ggn* in meiotic double-strand break repair and early embryonic development, the death of *Ggn* null mice during early embryogenesis precluded an analysis of its role in acrosome formation. GGN is localised in spermatocytes, spermatids and sperm tails in mouse and human testes (Jamsai *et al*., 2008). In addition, GGN is also known to interact with CRISP2 (Jamsai *et al*., 2008). This protein is incorporated into the developing acrosome and the outer dense fibres of the sperm tail (Foster and Gerton, 1996; O'Bryan *et al*., 2001). Knockout of *Crisp2* leads to sub-fertility in the mouse characterised by acrosome reaction defects and stiff mid-piece syndrome (Lim *et al*., 2019). It is thus plausible that in humans, GGN plays an as-yet unidentified role in acrosome formation. Interestingly, brothers GL-1 and GL-2 also harboured a missense variant in *PDCD2L* that was predicted to introduce a cryptic splice site in exon 4. As altered splicing could not be confirmed (data not shown), the gene is not enriched in the testis and a link to acrosome biology has never been reported, it is unlikely that this variant causes globozoospermia. The precise role of PDCD2L in spermatogenesis, or any other process, has not however, been tested.

Finally, we identified two variants in *DNAH17*. Parental samples were not available to test compound heterozygosity. A *Dnah17* knockout mouse is known to suffer from male infertility and abnormal hair growth (http://www.informatics.jax.org/). A more detailed description of the mouse spermatogenesis is currently not available. Recently, mutations in *DNAH17* were identified in patients with asthenozoospermia (Whitfield *et al*., 2019). It remains elusive whether the variants in *DNAH17* may cause infertility in GL-3.

### Acrosomal hypoplasia

The lack of acrosome in globozoospermic patients was originally attributed to absent formation, but Alvarez Sedo *et al*. (2012) documented deficiencies in a set of six subacrosomal proteins in globozoospermia and proposed that this may result in faulty acrosomal adherence to spermatid nuclei and subsequent acrosomal loss (Chemes, 2018). In addition to acrosomal absence, acrosomal hypoplasia (small, thin and detached acrosomes) may be present in up to 30–60% round or amorphous sperm heads of infertile men (Chemes, 2018). The correct identification of globozoospermia and acrosomal hypoplasia is very relevant because failed *in vitro* fertilisation due to low or absent Ca^2+^ oscillations following ICSI into oocytes is associated with defective acroplaxome, i.e. the cytoskeletal platform to which the acrosome normally adheres during spermatogenesis (Alvarez Sedo *et al*., 2012), discussed in (Chemes, 2018). As shown in the present report, there is not a clear-cut separation between a failure of acrosome biogenesis (globozoospermia) and failures of acrosome adhesion to the developing sperm head (acrosomal hypoplasia). Both conditions may coexist in the same semen sample.

### Genetic studies in globozoospermia

Recessive genetic disorders are common in inbred populations since in offspring born from consanguineous parents, stretches of the genome are homozygous as a result of inheriting identical chromosomal segments from both parents, which may carry rare variants that cause recessive disorders. Because consanguinity has previously been a common observation in globozoospermia cases (Dam *et al*., 2007; Harbuz *et al*., 2011; Koscinski *et al*., 2011), we used the exome data to identify stretches of homozygosity. In 7 out of 15 cases (47%), we studied through exome sequencing large (≥10-Mb) stretches of homozygosity were found, indicating consanguinity and again confirming that globozoospermia is more common in inbred populations (Table III, Supplementary Fig. S7 and Supplementary Table SV). In all seven cases, we identified a possible causative mutation in such regions, indicating that homozygosity mapping is an effective method to identify potential disease loci for globozoospermia.

In 7 out of 15 patients, no clear cause of globozoospermia could be identified. It is possible that our filtering strategy was too stringent and excluded the possibly pathogenic variants in the unsolved cases.

Patients with globozoospermia type I and wishing to conceive are forced to use ICSI for fertilisation. The success rate of fertilisation using ICSI for globozoospermic men is however lower than in unselected groups of male infertility (24.3% compared to 70–80%), and rates range between 0 and 100%, with most authors reporting low to no fertilisation (Dam *et al*., 2007; Palermo *et al*., 2009; Chansel-Debordeaux *et al*., 2015). This suggests that infertility in globozoospermia is more complex than just the presence or absence of the acrosome. As indicated above, this may be related to the presence of essential molecules required for oocyte activation in the acroplaxome. The fertilisation rate is restored by assisted oocyte activation (AOA) using mechanical, chemical and electrical approaches (Kuentz *et al*., 2013). The identification of the genetic cause of globozoospermia may help us to improve prediction of treatment success and may even impact future therapeutic strategies, which will aid in improving the clinical management of globozoospermia patients.

## Conclusion

Our study on globozoospermia patients identified homozygous nonsense mutations in human in two genes known to cause globozoospermia in mice, *ZPBP* and *CCDC62*. This definitively establishes the role of these genes in human globozoospermia. In addition, homozygous and heterozygous variants in five candidate novel genes for globozoospermia were identified. Elucidating the genetic cause of globozoospermia can help to better understand the aetiology of globozoospermia, which is invaluable to better understand the process of spermiogenesis and to predict the success as well as the risks of reproductive treatments such as ICSI.

## Supplementary Material

Suppl_fig1_dez246

Suppl_fig2_dez246

Suppl_fig3_dez246

Suppl_fig4_dez246

Suppl_fig5_dez246

Suppl_fig6_dez246

Suppl_fig7_dez246

Suppl_tableS1_dez246

Suppl_tableS2_dez246

Suppl_tableS3_dez246

Suppl_tableS4_dez246

Suppl_tableS5_dez246
